# Waterborne Protozoan Parasite and Thalassogenic Diseases in Marine Environment: Detection Techniques, Indicators and Public Health Implications

**DOI:** 10.3390/microorganisms14010098

**Published:** 2026-01-02

**Authors:** Pilar Suarez, José Luís Alonso, Gladys Vidal

**Affiliations:** 1 Environmental Engineering & Biotechnology Group (GIBA-UDEC), Environmental Science Faculty & EULA-CHILE Center, Universidad de Concepción, Concepción 4070386, Chile; pilarsuarez@udec.cl; 2 Water Research Center for Agriculture and Mining (CRHIAM), ANID Fondap Center, Victoria 1295, Concepción 4070411, Chile; 3 Laboratorio de Parasitología, Facultad de Ciencias Biológicas, Universidad de Concepción, Concepción 4070386, Chile; 4 Instituto de Ingeniería del Agua y Medio Ambiente, Universidad Politècnica de València, 46022 Valencia, Spain; jalonso@iiama.upv.es

**Keywords:** waterborne, *Cryptosporidium*, *Giardia*, *Blastocystis*, coastal area, seawater

## Abstract

Thalassogenic diseases are human infections associated with exposure to marine environments. This review explores the occurrence of *Cryptosporidium* spp., *Giardia duodenalis*, and *Blastocystis* sp. in seawater and shellfish and their implications for public health. Between 2015 and 2026, multiple studies reported the presence of these parasites in shellfish and seawater. *Cryptosporidium* spp. was found at average concentrations of 5.5 × 10^1^ oocysts/g in shellfish and up to 3.7 × 10^1^ oocysts/L in seawater. *Giardia duodenalis* reached 9.1 × 10^1^ cysts/g in shellfish, close to the infectious dose, and 3.5 × 10^1^ cysts/L in seawater. *Blastocystis* sp. showed prevalence rates of 33.82% in shellfish and 17.3% in seawater. These findings highlight a potential infection risk for bathers and seafood consumers, emphasizing the need to determine the specific species (or subtypes) involved and assess their viability to accurately evaluate public health implications. The persistence of these parasites in the environment needs improved monitoring. Future strategies should integrate next-generation sequencing (NGS) or use of various fecal indicators to enhance environmental surveillance and reduce health risks in coastal regions.

## 1. Introduction

Climate change is redesigning coastal ecosystems through rising sea temperatures, altered precipitation, and tidal changes, creating favorable conditions for the persistence and transmission of waterborne protozoan parasites [1,2]. These changes compromise seawater quality and increase human exposure risks via recreational activities and shellfish consumption [3,4].

Thalassogenic diseases, illnesses associated with marine environments, are responsible for over 120 million cases of gastroenteritis annually and 4 million cases of hepatitis A and E, with 40,000 deaths and permanent disabilities [5,6,7]. These diseases are strongly linked to microbial contamination of seawater, primarily caused by the discharge of untreated or inadequately treated sewage into marine ecosystems [3,4,8]. Protozoa such as *Cryptosporidium* spp., *Giardia duodenalis*, and *Blastocystis* sp. are of particular concern due to their capacity to cause gastrointestinal outbreaks. Between 2017 and 2022, 416 parasitic outbreaks were reported, with 75.2% linked to recreational water exposure and *Cryptosporidium* accounting for 77.4% [3].

These protozoan parasites represent a substantial global health burden, being associated with approximately 1.7 billion episodes of diarrhea and 842,000 deaths annually [9]. Their impact is particularly pronounced among children under five years of age and immunocompromised individuals, such as those living with HIV [10]. Infections can present a wide clinical spectrum, ranging from self-limiting acute diarrhea to persistent or chronic forms [10,11,12]. Furthermore, these organisms have been implicated as etiological agents in other conditions, including malabsorption syndrome, irritable bowel syndrome, cutaneous allergic reactions, and, in the case of *Cryptosporidium* spp., a potential link to colorectal cancer [9,10,11].

Despite growing evidence of protozoan presence in marine environments, their role in thalassogenic diseases remains underexplored, partly due to limitations in detection methods and biological factors that hinder diagnosis. Advancing and developing sensitive detection techniques, while integrating microbial indicators such as *Enterococcus* spp. with environmental parameters, is strongly recommended to improve surveillance and risk assessment. International guidelines, including those from WHO, emphasize the enumeration of *Enterococcus* in seawater [13]; however, this indicator has not been consistently adopted in some countries, such as Chile [14].

This article synthesizes current scientific knowledge on waterborne protozoan parasites in marine environments, emphasizing their role in thalassogenic diseases, detection methodologies, and the use of microbial indicators to support public health strategies.

## 2. Bibliometric Analysis

A comprehensive literature review was conducted using the Web of Science (WoS) database, focusing exclusively on peer-reviewed scientific articles published between 2015 and 2026. The search strategy incorporated the keywords “waterborne parasite”, “*Cryptosporidium*”, “*Giardia*”, and “*Blastocystis*”, in combination with the terms “shellfish”, “bivalve”, “clams”, “mussels”, “oyster”, “seawater”, and “coastal”. These terms were systematically combined across multiple search queries to optimize the retrieval of relevant publications. The search was refined to include only studies reporting the detection of parasites in marine environments, specifically in shellfish intended for human consumption and or seawater. Articles focusing on shellfish from estuarine or freshwater environments were excluded.

**Figure 1:**
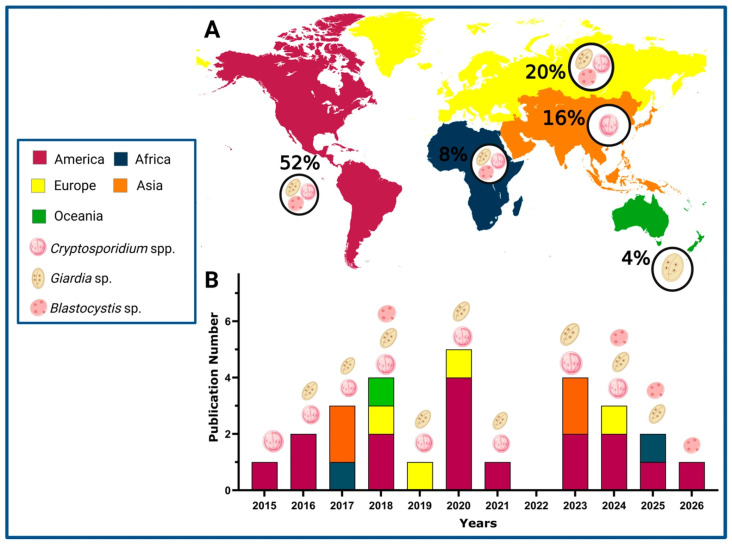
Geographical and temporal distribution of scientific publications on parasite detection in marine environment (2015–2026). (**A**) Percentage of geographical distribution by continents. (**B**) Temporal distribution (Created in https://Biorender.com, 10 October 2025).

A total of 25 articles were identified between 2015 and 2026 that reported the detection of waterborne parasites in marine environments (shellfish for human consumption and seawater) [15,16,17,18,19,20,21,22,23,24,25,26,27,28,29,30,31,32,33,34,35,36,37,38]. Figure 1 illustrates the geographical (Figure 1A) and annual (Figure 1B) distribution of scientific publications between 2015 and 2026. The Americas accounted for the highest proportion of studies (52%), particularly during 2018, 2020, and 2024. Europe and Asia also demonstrated substantial research activity, contributing 20% and 16% of the total publications, respectively, with notable increases in 2019 and 2023. Africa and Oceania contributed fewer studies, representing 8% and 4% of the total, respectively, although their presence remained consistent throughout the study period. The overall trend in publication volume was variable, with prominent peaks observed in 2018, 2020 and 2023.

Figure 1 also highlights the detection of *Cryptosporidium* spp. in 76%, *Giardia* sp. in 48%, and *Blastocystis* sp. in 16%. It is worth noting that the three parasites have been investigated in studies conducted across the Americas, Europe, and Africa. In contrast, research from Oceania and Asia has predominantly reported the presence of *Cryptosporidium* spp. These results reflect an increasing global focus on monitoring parasitic contamination in marine environments. Notably, *Blastocystis* sp. has gained particular attention in recent years, emphasizing its emerging relevance in marine parasitology and public health surveillance (Figure 1B).

## 3. Waterborne Parasite and Thalassogenic Disease

**Figure 2:**
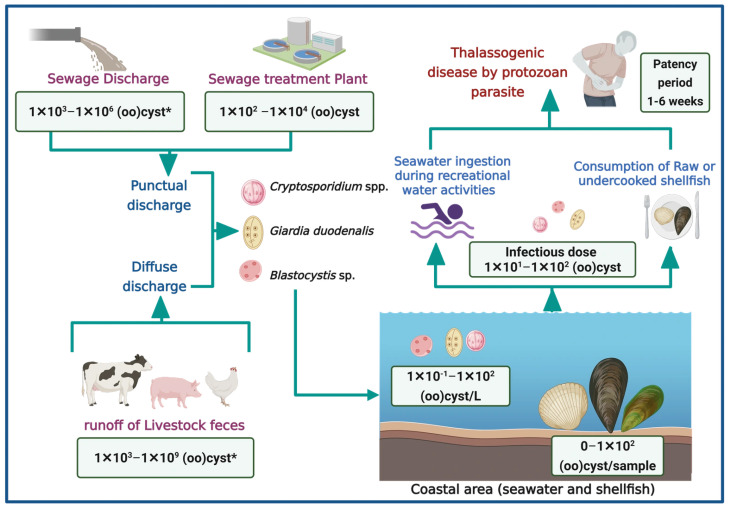
Pathways of waterborne parasite contamination in marine environment and associated health risk (Created in https://BioRender.com, 10 October 2025). (*): quantification in raw feces.

Figure 2 illustrates the main pathways through which *Cryptosporidium* spp., *G. duodenalis*, and *Blastocystis* sp. contaminate coastal waters, contributing to thalassogenic diseases. It highlights two primary sources of contamination: punctual and diffuse discharges of sewage discharges from sewage treatment plants and runoff from livestock feces [18,32,33,38,39,40]. These contaminants reach coastal zones, where they pose health risks through accidental ingestion during recreational water activities or via consumption of raw or undercooked shellfish.

It has been demonstrated that the concentration of (oo)cysts in human feces varies between 1 × 10^2^ and 1 × 10^6^ (oo)cyst/g [36,37]. Consequently, untreated sewage may reach similar concentrations. Furthermore, these parasites have been reported to exhibit resistance to chlorination and UV radiation disinfection, potentially allowing up to 1 × 10^4^ (oo)cysts to persist in treated effluents [41,42]. These discharges may significantly contribute to the presence of parasites in coastal environments. Given the zoonotic nature of many of these parasites, poor management of animal feces in extensive or subsistence livestock systems also plays a role in environmental contamination [43]. For instance, an animal infected with *Cryptosporidium* may excrete up to 1 × 10^9^ oocysts/g of feces [41,43]. The concentration of (oo)cysts in seawater and shellfish ranges from 0 to 1 × 10^2^, indicating a potential risk of infection for bathers or consumers [44].

Although there is limited information regarding outbreaks associated with recreational activities in marine waters or the consumption of shellfish, this gap may be attributed to several challenges inherent to waterborne parasitic infections. First, the incubation and patency period of these parasites is often prolonged defined as the interval between ingestion of the infective stage and the onset of symptoms, which can extend over several weeks, complicating the ability to establish a direct link between shellfish consumption and clinical manifestations (Figure 2) [45,46]. Parasites may remain detectable in clinical samples for extended periods, yet diagnostic techniques such as stool examinations require patients to be in the acute phase of infection to visualize parasitic elements effectively [41,45,46,47]. Given the detection threshold of approximately 1 × 10^4^ cysts/g, this leads to underreporting [47]. Finally, the absence of pathognomonic signs further hinders diagnosis, as these parasites typically do not produce specific or distinctive symptoms. Consequently, patients may receive only symptomatic treatment for sporadic diarrhea without etiological identification, potentially resulting in chronic infections or more severe clinical outcomes depending on the host’s immune status, nutritional condition, and age [41,45,46,47].

These factors may interfere with the integration of clinical and environmental data, limiting the ability to trace the origin of infections. Therefore, when reviewing a patient’s clinical history, it is essential to inquire about recent consumption of raw shellfish or exposure to coastal waters, even if such activities occurred up to two weeks prior. In suspected cases, parasitological analysis, particularly direct observation, should be included, as acute diarrheal episodes are associated with higher concentrations of (oo)cysts in feces (often exceeding 1 × 10^6^ (oo)cysts/g), increasing detection.

In this context, monitoring protozoan parasites in marine environments becomes a valuable tool for early warning systems, enabling timely public health interventions and outbreak mitigation strategies.

## 4. Methodological Approaches for the Detection of Waterborne Protozoan Parasites in Shellfish and Aquatic Environments

**Figure 3:**
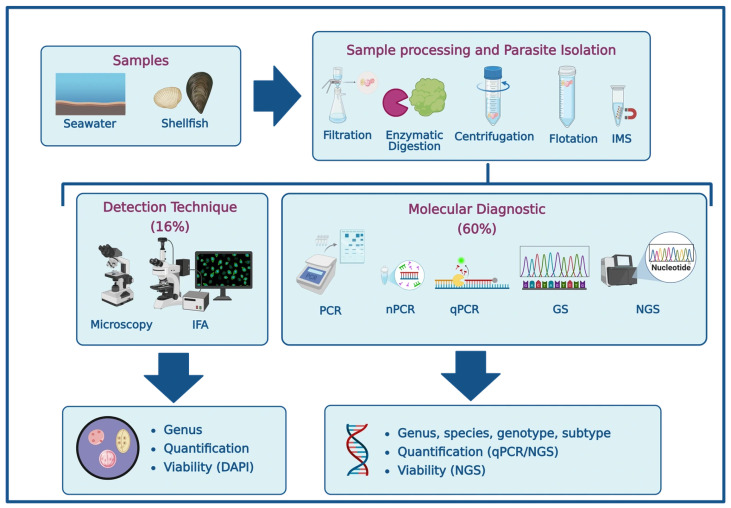
Methodological approaches for the detection of waterborne protozoan parasites in marine environments (Created in https://BioRender.com, 10 October 2025). [IMS]: Immunomagnetic separation; [IFA]: Immunofluorescence assay; [PCR]: Polymerase chain reaction; [nPCR]: nested PCR; [qPCR]: quantitative PCR; [GS]: Gene sequencing; [NGS]: Next-generation sequencing; [DAPI]: 4′,6-diamidino-2-phenylindole.

Among the 25 scientific articles reviewed, a wide range of detection methods was reported [15,16,17,18,19,20,21,22,23,24,25,26,27,28,29,30,31,32,33,34,35,36,37,38]. Despite the growing availability of advanced molecular tools, microscopy remains a relevant and widely used screening technique for protozoan parasites in environmental samples. As shown in Figure 3, 16% of the studies relied solely on microscopic methods, such as optical microscopy (20%) and immunofluorescence assays (IFA, 40%), while 60% employed molecular techniques and 24% combined both approaches. Conventional PCR and nested PCR (nPCR) were the most frequently used molecular methods (68%), followed by gene sequencing (64%) and quantitative PCR (qPCR, 32%). Notably, only one study utilized Oxford Nanopore sequencing technology [15,16,17,18,19,20,21,22,23,24,25,26,27,28,29,30,31,32,33,34,35,36,37,38].

Detection strategies varied depending on the biological matrix (e.g., shellfish tissue vs. seawater), the target organism, and the analytical resolution required (e.g., presence/absence vs. species or genotype identification). Importantly, shellfish such as clams, mussels, and oysters not only act as potential transmission vectors but also serve as bioindicators of marine water quality, concentrating protozoan (oo)cysts from surrounding waters and thereby facilitating environmental surveillance [48,49,50,51].

### 4.1. Sample Processing and Parasite Isolation

In shellfish, parasite recovery typically involves mechanical or enzymatic disruption of digestive tissues, followed by concentration and purification steps. Common techniques include:

• Filtration: Membranes with 0.2 µm pore sizes retain protozoan (oo)cysts (0.2–20 µm). Polycarbonate and cellulose nitrate membranes are selected based on charge compatibility with *Cryptosporidium* oocysts and downstream molecular applications [48,52].
• Enzymatic digestion: Pepsin or proteinase K is used to release (oo)cysts from lipid and glycoprotein-rich tissues, which may interfere with detection. Hemolymph components can also affect immunofluorescence assays due to background fluorescence [48,49,51,53].
• Centrifugation and flotation: Cesium chloride gradients improve (oo)cyst recovery compared to sucrose, though both may coextract similar density particles [48].
• Immunomagnetic separation (IMS)**:** Applied mainly for *Cryptosporidium* and *Giardia*, IMS uses monoclonal antibodies to isolate (oo)cysts, with better performance in water samples due to lower matrix complexity [48,49].

Ligda et al. [49] reported recovery rates of 32.1% for *Giardia* and 61.4% for *Cryptosporidium* in *Mytilus galloprovincialis* using pepsin digestion and IMS, with a detection limit of 10 (oo)cysts/g. Bigot-Clivot et al. [50] showed that *M. edulis* bioaccumulates these parasites for up to 21 days post-depuration, supporting its use as a sentinel species. Shellfish serve both as transmission vectors and bioindicators, concentrating protozoan (oo)cysts from surrounding waters and enabling integrated food safety and environmental monitoring [49,50].

In seawater, isolation methods include membrane filtration for environmental DNA, ultrafiltration and PEG precipitation for parasite concentration, and modified CTAB protocols for nucleic acid extraction with inhibitor removal [54].

The concentration of parasitic protozoa in environmental samples, such as water or shellfish, is low (generally <100 (oo)cysts) [48]. Moreover, the presence of interfering substances in these matrices could be compromise downstream analyses, including PCR [48,49,50]. Sample processing and isolation methods may be applied individually or in combination, depending on matrix characteristics [51]. For instance, Kim et al. [51] reported that, for detecting parasites in shellfish, immunomagnetic separation (IMS) significantly enhances sensitivity, enabling detection of fewer than 10 oocysts per tissue for both *C. parvum* and *G. duodenalis*. Additionally, incorporating enzymatic digestion further improves sensitivity, reaching levels as low as 5 oocysts/tissue [48,51,53].

### 4.2. Detection Techniques

Detection of waterborne protozoa typically begins with:

• Microscopy: Techniques such as Ziehl–Neelsen staining, DAPI fluorescence, and differential interference contrast allow genus-level identification and viability assessment, though sensitivity is limited and influenced by sample quality [47].
• IFA: Monoclonal antibodies conjugated to fluorophores are used for species-level identification and quantification [47,48,50].

While microscopy remains a classical tool for preliminary screening, its limitations are evident in marine matrices [48,49,50,51]. Interfering substances in seawater and shellfish can lead to false positives or negatives, and low parasite concentrations (often ~10^2^ oocysts) fall below the detection threshold of conventional microscopy (~10^4^ oocysts), potentially resulting in underestimation of contamination levels. Its importance of selecting appropriate sample preparation and detection techniques based on the matrix and target parasite [50].

### 4.3. Molecular Diagnostics and Target Genes

Molecular techniques offer high sensitivity and specificity, detecting as few as 1–5 (oo)cysts/sample [55]. Common methods include:

• Conventional PCR: Genus-level detection.
• nPCR: Enhanced sensitivity for species/subtype identification.
• qPCR: Real-time detection and quantification.
• Target Gene Sequencing: Subtype confirmation.

Frequently targeted genes include *gp60* and COWP for *Cryptosporidium*, *β-giardin*, *gdh*, and *tpi* for *G. duodenalis*, and SSU rRNA for *Blastocystis* subtype identification [47,56,57]. Although molecular methods reduce subjectivity, challenges remain due to matrix inhibitors and mixed infections. In this context, molecular technologies offer enhanced sensitivity and improved specificity, given that their performance does not rely on the subjective interpretation of the technician [47,55]. Next-generation sequencing (NGS) technologies offer improved resolution for complex samples [58].

### 4.4. Next-Generation Sequencing

Detecting protozoan pathogens in aquatic environments is challenging due to their low abundance, genetic diversity, and complex sample matrices. In this context, next-generation sequencing (NGS), particularly 18S rRNA metabarcoding and shotgun metagenomics, has emerged as a powerful tool for environmental and food safety surveillance [58,59,60,61,62].

DeMone et al. [59] developed a metabarcoding assay targeting the V4 region of the 18S rRNA gene for detecting *Cryptosporidium parvum*, *Giardia enterica*, and other protozoa in oysters. While the assay enabled multi species detection and showed higher sensitivity than conventional PCR in tissue samples, host DNA interference reduced its performance, especially for *Giardia* in hemolymph. Mitigation strategies included pepsin-HCl digestion and synthetic gBlocks.

Although NGS has not yet been applied to marine water for protozoan detection, studies in sewage environments demonstrate its utility. Zahedi et al. [60] used 18S rRNA metabarcoding and targeted NGS to analyze samples from sewage treatment plants in Australia. General 18S NGS detected *Blastocystis* sp., while targeted assays identified nine *Cryptosporidium* species, including five zoonotic types. A broader survey across 25 treatment plants revealed 17 *Cryptosporidium* species and six genotypes, with oocyst concentrations ranging from 7.0 × 10^1^ to 1.8 × 10^4^ oocysts/L, highlighting spatial and seasonal diversity [60,61].

In South Africa, Mthethwa et al. [62] applied 18S rRNA amplicon sequencing and shotgun metagenomics to treated and untreated sewage. They identified *Cryptosporidium* spp. (3.48%), *Blastocystis* sp. (2.91%), and *Giardia intestinalis* (0.31%), along with virulent genes and metabolic pathways, suggesting protozoan viability within treatment systems.

In summary, NGS enables sensitive, multi-target detection and functional profiling of protozoan parasites. While its application is limited by cost, technical complexity, and host DNA interference, it complements conventional methods and enhances microbial risk assessment when combined with optimized sample preparation strategies [58,59,60,61,62].

## 5. Fecal Indicators in Seawater Associated with Waterborne Parasite

Due to the complexity and cost of targeted parasitological diagnostics, microbial indicators offer a practical alternative for preliminary assessment of protozoan contamination in seawater. Table 1 summarizes commonly used indicators, including *Escherichia coli*, *Enterococcus* spp., total and thermotolerant coliforms, *Clostridium perfrigens*, bacteriophages, and Pepper mild mottle virus (PMMoV), with the latter used in microbial source tracking [8,31,33,63,64,65,66].

While correlations between bacterial indicators and protozoa have been explored in freshwater and sewage systems, results are inconsistent. In marine environments, a two-year study on Brazilian beaches found no significant correlation between bacterial indicators and the presence of *Cryptosporidium* spp. or *Giardia* sp. (Pearson’s *p* ≤ 0.05). Parasite concentrations varied seasonally, with *Cryptosporidium* ranging from 2.1 to 7 oocysts/L and *Giardia* from 5.7 to 9.3 cysts/L, while bacterial counts were generally lower during parasite detection periods [33].

In contrast, Graczyk et al. [8] reported strong positive correlations between *Enterococcus* spp. and both *C. parvum* (R = 0.95; *p* < 0.01) and *G. duodenalis* (R = 0.71; *p* = 0.018) in recreational marine waters, suggesting its potential as a predictive marker.

González-Fernández et al. [31] linked microbial indicators with environmental parameters such as rainfall, temperature, and salinity. In Costa Rica, *Cryptosporidium* was detected in 33% and *Giardia* in 11% of samples during the rainy season, associated with temperatures of 27–28.8 °C and salinities of 27.6–31.4 ppt. Turbidity showed no correlation.

Among all indicators, *Enterococcus* spp. showed the highest predictive value for protozoan presence, with reported sensitivity of 88%, specificity of 93%, positive predictive value (PPV) of 78%, and negative predictive value (NPV) of 96% [31].

Anaerobic sulphite-reducing Clostridia, particularly *Clostridium perfringens* spores, have been proposed as useful surrogate indicators for the presence or persistence of *Cryptosporidium* oocysts in water (Table 1). Their resistance to environmental stressors and disinfection processes parallels that of protozoan (oo)cysts, making them valuable for assessing treatment efficacy and potential contamination risks. Several studies have demonstrated correlations between *C. perfringens* counts and the occurrence or removal of protozoan pathogens, including *Cryptosporidium* [63,64,65,66]. However, while *C. perfringens* spores can indicate the potential for contamination by similarly resistant microorganisms, they should be regarded as surrogate or complementary indicators, not direct markers of *Cryptosporidium* presence.

The measurement of bacterial indicators, as presented in Table 1, is commonly performed through culture-based methods followed by quantification (by MPN). This approach offers several advantages over routine parasitological detection techniques. Bacterial culturing is relatively simple, cost-effective, and widely standardized, making it more accessible for routine monitoring programs. In contrast, the detection of protozoan parasites such as *Cryptosporidium* and *Giardia* typically requires molecular techniques that are more complex, expensive, and technically demanding, often involving specialized equipment and trained personnel.

Given their simplicity and cost-effectiveness, bacterial indicators quantified via culture-based methods are suitable for routine monitoring. While not definitive, they provide a useful screening tool to guide targeted parasitological analyses, especially during high-risk periods such as summer or in shellfish harvesting areas. In such contexts, water monitoring should precede parasite detection, and shellfish intended for consumption should undergo microscopic examination for parasitic stages.

## 6. Waterborne Protozoan Parasites in Marine Environment

Several studies have confirmed the presence of waterborne parasites in marine environments, particularly in shellfish intended for human consumption and seawater [15,16,17,18,19,20,21,22,23,24,25,26,27,28,29,30,31,32,33,34,35,36,37,38]. These studies have analyses various species of clams, including *Mya arenaria*, *M. truncata*, and *Ruditapes decussatus*; mussels such as *Aulacomya atra*, *Geukensia demissa*, *Mytilus* spp., *M. edulis*, *M. galloprovincialis*, *Perna canaliculus*, *P. perna* and *P. viridis*; and oysters belonging to the genera *Crassostrea* spp., *Crassostrea virginica*, *C. belcheri*, *C. lugubris C. iredalei*, *Pinctada radiata*, *Argopecten irradians*, *Saccostrea forskali* and *Venerupis philippinarum.* Table 2 provides a summary of findings related to the detection of *Cryptosporidium* spp., *Giardia duodenalis*, and *Blastocystis* sp. in marine environmental between 2015 and 2026.

*Cryptosporidium* spp. has been identified in shellfish intended for human consumption, with multiple species and subtypes reported, including *C. hominis* (IbA10G2R2), *C. parvum* (IaA15G2R1), *C. meleagridis* and *C. andersoni* [15,16,17,18]. Among the identified species, *C. hominis* subtype IbA10G2R2 (classified based on the *gp60* gene) has been exclusively detected in humans [57]. Additionally, *C. parvum* and *C. meleagridis* are recognized as zoonotic species [41]. Notably, *C. parvum* subtype IaA15G2R1 is the most frequently reported subtype associated with outbreaks linked to contaminated water and food, with transmission routes involving both animal and human fecal contamination [57]. As shown in Table 2, the average concentration of oocysts detected in shellfish samples was 5.5 × 10^1^ oocysts/sample. Infective doses for *Cryptosporidium* spp. have been reported to range between 1 × 10^1^ and 1 × 10^2^ oocysts [41]. This implies a potential health risk for the population, as the number of oocysts found in shellfish falls within the infective dose range for this parasite. Prevalence rates varied significantly depending on the detection technique adopted. Microscopic techniques yielded an average prevalence of 24.5%, meanwhile molecular techniques reported a lower average prevalence of 10.53% (Table 2). In seawater samples, *C. parvum* was predominant species detected, with concentrations reaching up to 3.7 × 10^1^ oocyst/L. Prevalence in seawater average 19% using microscopic methods and approximately 10% when assessed by molecular techniques [29,30,31,32].

Regarding *G. duodenalis*, assemblages A, AI, AII, B, C, and D have been identified in shellfish. Assemblages A and B are considered zoonotic, yet they have also been observed in humans at rates of 64% and 72%, respectively [67]. In contrast, assemblages C and D are primarily associated with canine hosts, with detection rates of 87% and 94%, respectively [41]. As shown in Table 2, the prevalence of *G. duodenalis* in shellfish ranged from 7%. Notably, concentrations in shellfish reached up to 9.1 × 10^1^ cysts/g. Regarding its detection in seawater, assemblage AII has been identified, with mean concentrations of 3.5 × 10^1^ cyst L^−1^ and prevalence rates 21%. In shellfish, the reported concentration exceeds the infectious dose for this parasite, estimated to range between 1 × 10^1^ and 1 × 10^2^ cyst [41].

*Blastocystis* sp. has been detected in shellfish with a wide diversity of subtypes, including ST3, ST7, ST14, ST23, ST26, and ST44 [18,34,35,36]. In seawater samples, subtypes ST1, ST2, ST3, and ST10a have been identified [37,38]. Although *Blastocystis* sp. is considered zoonotic, subtypes ST1–ST9 are frequently found in humans, with ST3, ST1, and ST2 being the most prevalent, accounting for over 90% of cases [68,69]. In contrast, subtypes ST7, ST10, ST14, ST23, and ST26 are more commonly associated with animal hosts such as birds, dogs, cattle, pigs, and sheep, with prevalence rates ranging from 50% to 90% in these animals [68]. These findings may suggest both point-source and diffuse fecal contamination in the areas where the studies were conducted. Although quantitative concentrations were not reported, prevalence rates reached 34% in shellfish and 17% in seawater [18,34,35,36,37,38].

As shown in Table 2, the identification of parasitic species is essential for determining the source of fecal contamination; nevertheless, it is a process that relies on molecular techniques. Additionally, quantifying waterborne parasites in environmental samples is necessary to assess public health risks, typically achieved through microscopy or qPCR. It is also important to include the assessment such as DAPI (4′,6-diamidino-2-phenylindole), for (oo)cyst viability, as it provides crucial information about the potential infectivity of parasites. Although molecular methods are powerful tools for detection and identification, their applicability in rural areas may be limited due to infrastructure and resource restrictions. These findings highlight the urgent need to strengthen environmental surveillance systems and sanitary controls in coastal areas.

## 7. Conclusions and Future Perspective

Coastal environments act as reservoirs and transmission routes for waterborne protozoan parasites such as *Cryptosporidium* spp., *Giardia duodenalis*, and *Blastocystis* sp., posing a public health risk through recreational water exposure and shellfish consumption. Detection variability across methodologies highlights the need for integrated diagnostic approaches.

Next-generation sequencing (NGS) enhances sensitivity and specificity, while microbial indicators, combined with physicochemical parameters, offer cost-effective preliminary screening. Strengthening local laboratory capacities through training programs, mobile diagnostic units, and investment in basic infrastructure is crucial to ensure equitable access to parasitological surveillance in rural coastal communities. Seasonal monitoring in high-risk areas should include both water and shellfish matrices to support comprehensive risk assessment and One Health strategies.

## Figures and Tables

**Table 1:** Fecal indicator for assessing microbiological quality of seawater and their association with the presence of *Cryptosporidium* spp. and *Giardia* sp.

Fecal Indicator	Specie	Detection Technique	Association	Reference
Bacteria	Total coliform	C	N	[31]
	*Escherichia coli*	C	N	[33]
	*Enterococcus* spp.	C	+	[31,33]
			N	[8]
	*Clostridium perfrigens*	C	+	[63,64,65,66]
Virus	Bacteriophages	C/qPCR	+	[31]
	PMMoV *	RT-PCR	+	[31]

* PMMoV: Pepper mild mottle virus; [C]: cultivation; [PCR]: Polymerase chain reaction; [qPCR]: quantitative PCR; [RT-PCR]: Real-Time PCR; [N]: No correlation; [+]: positive correlation.

**Table 2:** Evidence of Waterborne parasites in shellfish and seawater (2015–2026).

Waterborne Parasite	Type of Sample	Specie or Genotype or Suptype Detected	Concentration Reported	Detection Technique	Prevalence (%)	Reference
*Cryptosporidium* spp.	Shellfish (Mussel, Clams and Oyster)	*C. hominis* (IbA10G2R2, IfA19G1), *C. parvum* (IIaA15G2R1, IIaA11G2R2, IIaA15G2R2, IIaA16G3R1, IIaA17G2R1, IIaA19G2R2, IIaA20G2R2, IIaA20G3R2), *C. meleagridis*, *C. andersoni*	5.5 × 10^1^ * (0–1.3 × 10^1^)	ZNS/IFA	26 (14–40)	[15,16,17,18]
				PCR/nPCR/RFLP/qPCR/GS	13 (0–36)	[15,19,20,21,22,23,24,25,26,27,28,29]
	Seawater	*C. parvum*	3.7 × 10^1 +^ (1.1 × 10^−1^–2.8 × 10^2^)	ZNS/IFA	19 (0–29)	[30,31,33]
				PCR	10 (0–27)	[32,33]
*Giardia duodenalis*	Shellfish (Mussel, Clams and Oyster)	AI, AII, B, C, D	9.1 × 10^1^ ** (0–3.9 × 10^2^)	IFA nPCR/RFLP/ qPCR	7 (0–30)	[20,21,22,23,24,25,27,34]
	Seawater	AII	3.5 × 10^1 ++^ (1.0 × 10^−1^–1.1 × 10^2^)	IFA	21 (6–38)	[30,31,33]
				PCR	-	[33]
*Blastocystis* sp.	Shellfish (Mussel)	ST3, ST7, ST14, ST23, ST26, ST44	N.D.	PCR/qPCR/GS	34 (10–62)	[18,34,35,36]
	Seawater	ST1, ST2, ST3, ST10a	N.D.	PCR/GS	17 (0–50)	[37,38]

[ZNS]: Ziehl–Neelsen Stain; [IFA]: Immunofluorescence Antibody; [PCR]: Polymerase chain reaction; [nPCR]: nested PCR; [RFLP]: Restriction Fragment Length Polymorphism; [qPCR]: Quantitative PCR; [GS]: Gene Sequencing; [*]: oocyst/sample; [**]: cyst/sample; [^+^]: oocyst/L; [^++^]: cyst/L; [N.D.]: Not Determined.

## Data Availability

No new data were created or analyzed in this study. Data sharing is not applicable to this article.
